# Anti-Inflammatory and Organ-Protective Effects of Resveratrol in Trauma-Hemorrhagic Injury

**DOI:** 10.1155/2015/643763

**Published:** 2015-07-26

**Authors:** Fu-Chao Liu, Yung-Fong Tsai, Hsin-I Tsai, Huang-Ping Yu

**Affiliations:** ^1^Department of Anesthesiology, Chang Gung Memorial Hospital, Taoyuan City 333, Taiwan; ^2^College of Medicine, Chang Gung University, Taoyuan City 333, Taiwan; ^3^Graduate Institute of Clinical Medical Sciences, Chang Gung University, Taoyuan City 333, Taiwan

## Abstract

Resveratrol, a natural polyphenolic compound of grape and red wine, owns potential anti-inflammatory effects, which results in the reduction of cytokines overproduction, the inhibition of neutrophil activity, and the alteration of adhesion molecules expression. Resveratrol also possesses antioxidant, anti-coagulation and anti-aging properties, and it may control of cell cycle and apoptosis. Resveratrol has been shown to reduce organ damage following traumatic and shock-like states. Such protective phenomenon is reported to be implicated in a variety of intracellular signaling pathways including the activation of estrogen receptor, the regulation of the sirtuin 1/nuclear factor-kappa B and mitogen-activated protein kinases/hemeoxygenase-1 pathway, and the mediation of proinflammatory cytokines and reactive oxygen species formation and reaction. In the recent studies, resveratrol attenuates hepatocyte injury and improves cardiac contractility due to reduction of proinflammatory mediator expression and ameliorates hypoxia-induced liver and kidney mitochondrial dysfunction following trauma and hemorrhagic injuries. Moreover, through anti-inflammatory effects and antioxidant properties, the resveratrol is believed to protect organ function in trauma-hemorrhagic injury. In this review, the organ-protective and anti-inflammatory effects of resveratrol in trauma-hemorrhagic injury will be discussed.

## 1. Introduction

**Figure 1:**
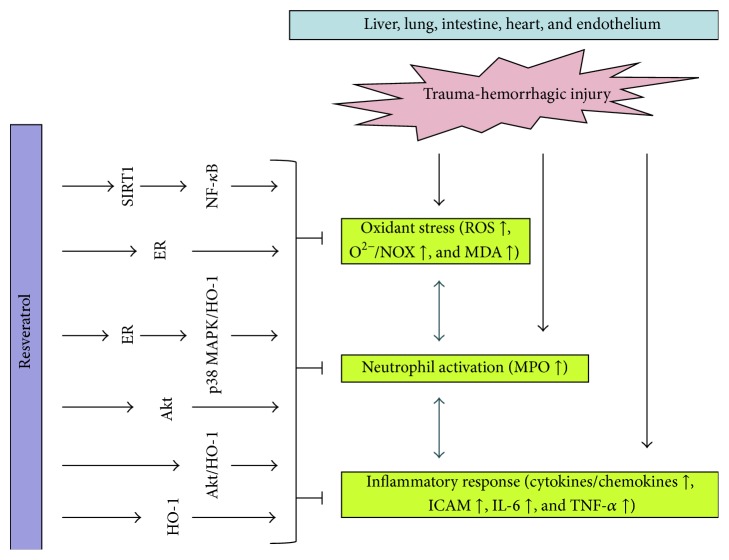
The anti-inflammatory and protective pathways of resveratrol in trauma-hemorrhagic injury. ER: estrogen receptor; SIRT1: sirtuin 1; HO-1: hemeoxygenase-1; p38 MAPK: p38 mitogen-activated protein kinase; NF-*κ*B: nuclear factor-kappa B; ROS: reactive oxygen species; MDA: malondialdehyde; NOX: NADPH oxidase; MPO: myeloperoxidase; ICAM: intercellular adhesion molecule; IL-6: interleukin 6.

Resveratrol is a naturally occurring plant antibiotic known as phytoalexins, found in various plants and fruits, especially abundant in grapes and red wine [1, 2]. Previous reports have demonstrated the protective effects of resveratrol in different pathological conditions and experimental models [3–7]. Many clinical studies also indicated the beneficial effects of resveratrol in human diseases [8–13]. A growing body of evidence showed that resveratrol might play potential therapeutic roles in human health by its anti-inflammatory, antioxidant, antiaging, antidiabetic, anticoagulative, and apoptotic properties [1, 7, 14, 15]. Resveratrol attenuates organ injury in trauma-hemorrhagic (T-H) injury through multiple pathways, in which a number of the molecular targets and protective effects of resveratrol have been identified, including the estrogen receptors (ER) [16, 17], the protein kinase B (Akt), the AMP-activated protein kinase (AMPK) [18, 19], the hemeoxygenase-1 (HO-1) [20–22], the histone/protein deacetylase sirtuin 1 (SIRT1) [23, 24], and nuclear factor-kappa B (NF-*κ*B) [25, 26] (Figure 1).

A variety of laboratory and clinical researches also show that resveratrol may lead to tissue- and organ-protective effects against various injuries [27–33]. Traumatic injury is recognized to induce the excessive production of oxidants and proinflammatory mediators and subsequent development of multiple organ dysfunctions [34–37] and resveratrol has been suggested to have organ-protective effect on trauma and hemorrhagic injuries due to its antioxidative activities and anti-inflammatory effects [18, 20, 38–42]. Trauma-hemorrhagic injury causes excessive production of proinflammatory mediators, cytokines, and chemokines. The enhanced secretion of proinflammatory cytokines is a critical factor in the initiation and perpetuation of organ injury [39, 43, 44]. These cytokines recruit other immune cells including neutrophils, thereby increasing leukocyte activation and trafficking result in organ injury [45–47]. In this review, we summarize the protective effects and possible mechanisms of resveratrol on the preservation of organ function in T-H injury (Table 1).

## 2. The Pulmonary Protective Effect of Resveratrol in T-H Injury

The activation of neutrophils in T-H injury [39, 47–49] and pulmonary injury is associated with an increased neutrophil accumulation [40, 42]. Neutrophils leave the microcirculation and migrate to matrix proteins or other cells and release mediators, which diffuse across the endothelium and hurt parenchymal cells [46, 47]. The intercellular adhesion molecule 1 (ICAM-1), constitutively present on the surface of endothelial cells, enhances firm adhesion of neutrophils to the vascular endothelium and is markedly upregulated following T-H injury [40, 47, 50]. For example, pulmonary ICAM-1 expression is increased in the lung in T-H shock [40, 47, 51]. The activated neutrophils appear to infiltrate the injured lung in parallel with increased expression of adhesion molecules on endothelial cells and also lead to the elevation of local chemokines/cytokines [40, 47, 51]. Chemotaxis has an important functional response to chemokines and is a key event in the recruitment of neutrophils in inflammation. Cytokine-induced neutrophil chemoattractant 1 (CINC-1) and CINC-3 are members of the IL-8 family and are potent chemoattractants for neutrophils [39, 42]. Moreover, the levels of the CINC-1 and CINC-3 are elevated in T-H injury. Furthermore, convincing evidence has shown that interleukin 6 (IL-6) plays an important role in organ injuries and is required for the expression of adhesion molecules and release of chemokines [52–54]. IL-6-deficient mice show less neutrophils infiltration and organ damage as compared with wild-type mice under hemorrhagic shock [54]. IL-6 could be released from macrophages and lymphocytes and appears to be an essential component of the inflammatory cascade that is associated with organ damage in T-H injury [51, 55].

Resveratrol has a protective role in organ damage following T-H injury via the reduction of neutrophil accumulation [46, 47, 49]. The role of resveratrol in the attenuation of lung injury is likely due to the reduction of chemokines in T-H injury [40, 42, 51]. IL-6, a critical early mediator in the lung during T-H injury, is inhibited by resveratrol treatment [42, 51]. The ability of resveratrol to modulate the expression of inflammatory cytokines, adhesion molecules, and chemokines suggests a role for resveratrol in the regulation of lung inflammation.

## 3. The Liver Protective Effect of Resveratrol in T-H Injury

The liver is considered to be a critical organ in the development of delayed organ dysfunction in patients having traumatic injuries and hemorrhagic shock [37, 39, 45, 56]. T-H injury results in massive production of proinflammatory mediators (IL-6, ICAM-1, CINC-1, and CINC-3) and the subsequent accumulation of neutrophils in the injured liver [39, 41, 47, 48]. Resveratrol reduces cytokine production and neutrophil accumulation in a rodent model of LPS-induced hepatic oxidative stress and inflammation [57].

Resveratrol binds to ER-*α* and ER-*β* and therefore alters the transcriptional activity of estrogen-responsive target genes [17, 19, 58, 59]. Resveratrol could modulate TNF-*α* genes expression and suppress IL-6 transcription via an ER-*α* signal integration [16]. Other studies demonstrated the role of sexual dimorphism in response to injury and showed the importance of sex steroids on the maintenance of organ function in T-H injury [45, 47, 60, 61]. The administration of resveratrol in combination with an ER antagonist ICI 182,780 blocks the hepatoprotective effect and such ER pathway is critical in hepatoprotection in T-H injury [41]. Building on these findings, ER pathways may be potentially useful therapies in the treatment of trauma patients [41, 62, 63]. In addition, estrogen treatment upregulates phosphatidylinositol 3-kinase (PI3K)/Akt expression via an estrogen receptor following T-H injury [64].

HO-1, a stress-inducible heme-degrading enzyme, provides cytoprotection against oxidative stress and inflammatory reaction [65, 66]. HO-1 expression is upregulated during T-H injury, and its induction appears to play a central role in the preservation of organ microcirculation under such conditions [67, 68]. A growing body of evidence demonstrates that Akt activation induces HO-1, which is known to have a protective effect in many organs under various deleterious conditions, including T-H injury [39, 42, 68, 69]. The upregulation of HO-1 causes a reduction of chemokines, cytokines, and adhesion molecules. It also decreases neutrophil accumulation and ameliorates organ injury in trauma-related shock status [39, 42, 70]. The administration of 17*β*-estradiol or flutamide (an antiandrogen drug) in T-H injury increases HO-1 expression, which attenuates the organs' dysfunction and injury [67–69]. The PI3K/Akt is an important signaling pathway controlling endogenous negative feedback or compensatory mechanism, in which proinflammation and chemotactic events are limited in response to injury [37, 39, 64]. Activation of PI3K/Akt signaling cascade by resveratrol has been observed in different tissues [38, 39, 71, 72]. Resveratrol-mediated increase in HO-1 is found to be Akt-dependent. When resveratrol is coadministered with PI3K/Akt antagonist, it abolishes the resveratrol-mediated HO-1 increase and hepatic protective effects in T-H injury [39, 70]. These results indicate that resveratrol attenuated liver damage and decreased proinflammatory mediator expression in T-H injury, likely through Akt-dependent HO-1 pathway [39, 70].

In addition, Powell et al. demonstrated that resveratrol could reduce T-H injury-induced mitochondria damage and hepatocyte injury. Resveratrol illustrates a protective effect through an increase in SIRT1 expression and a decrease in p53 and NF-*κ*B activity. It also inhibits proinflammatory mediator IL-6 and lipoperoxidation MDA expression [73].

## 4. The Intestinal Protective Effect of Resveratrol in T-H Injury

Intestinal tract is highly sensitive to injury. T-H injury could induce oxidants release, leading to microvascular permeability change, interstitial edema, mucosal barrier dysfunction, and inflammatory cell infiltration. ER also plays a pivotal role in intestinal injury after T-H shock. Previous reports showed that ER leads to the induction of p38 MAPK [18, 47, 74, 75], which contributes to the protection of cell/tissue in response to a variety of stimuli [76–78]. Estrogen-mediated anti-inflammatory and organ-protective effects are abolished by the administration of a p38 MAPK inhibitor SB-203580 following T-H injury [18, 75].

P38 MAPK activation regulates mucosal recovery in ischemic-injured porcine ileum [79] and protects glomerular epithelial cells against complement-mediated cell injury [80]. The p38 MAPK phosphorylation contributes to intestine-protection in T-H injury after ischemic preconditioning or T-H [18, 47, 74, 75]. Resveratrol also reduces chronic colonic inflammation [81] and protects H_2_O_2_-treated embryonic rat heart H9c2 cells via the p38 MAPK pathway [82].

The upregulation of HO-1 is known for its protective role in cellular stress during inflammation, ischemia, and radiation, as well as the anti-inflammatory and antiapoptotic effects. Resveratrol protects the intestinal epithelial barrier function against TNF-*α* and oxidative stress through upregulation of HO-1 expression in intestinal ischemia/reperfusion injury [83]. p38 MAPK activation induces HO-1 expression and maintains organ function under various stresses and injuries. Recent studies indicate that the treatment of animals with SB-203580, which blocks p38 MAPK, abolishes resveratrol-induced upregulation of HO-1 after T-H [18, 75]. These findings indicate that the salutary effects of resveratrol-mediated attenuation of intestinal injury in T-H are mediated, at least in part, through ER-dependent p38 MAPK/HO-1 upregulation.

## 5. The Cardioprotective Effect of Resveratrol in T-H Injury

Resveratrol has been shown to possess cardioprotective effects during ischemia-reperfusion injury [84, 85] and decreases organ injury in T-H injury [38, 86]. Cardiac injury is associated with increased neutrophil accumulation [38, 47] and such in small intestine is correlated with the attenuation of trauma-hemorrhage-induced cardiac dysfunction [18].

Activation of the PI3K pathway protects cells or organs against hypoxia and ischemia-reperfusion injury via inhibition of the apoptosis machinery [87, 88]. Modulation of the PI3K/Akt pathway with the PI3K inhibitor wortmannin suppresses coagulation and inflammation and decreases the survival of mice subjected to sepsis [89]. PI3K/Akt pathway also mediates neutrophils activation and regulates leukocyte signaling and function, to undergo chemotaxis [90]. Resveratrol decreases the production of proinflammatory mediators and ameliorates cardiac injury in T-H injury [38]. Blockade of Akt activation abolishes the salutary effects of resveratrol in the heart following T-H [38]. Altogether, resveratrol-related cardioprotective effect is likely mediated through an Akt-dependent pathway in T-H injury [38].

SIRT1 has been shown to regulate the mammalian genes transcription and silence the tumor suppressor genes [91, 92]. The SIRT1 transcription-modulating proteins demonstrate a fine balance in response to intracellular stimulus, such as hypoxia or stress signals. The beneficial effects of resveratrol mediated by SIRT1 activation can be contributed by different organs [24, 86, 93, 94]. Resveratrol improves heart function following T-H injury by downregulating SIRT1 expression [86]. The protective effect of resveratrol on left ventricular contractility and systemic TNF-*α* levels is abolished by sirtinol (a SIRT1 inhibitor) [86]. In addition, Jian et al. indicated that SIRT1 enzyme activity is decreased following T-H injury [94]. SIRT1 modulates left ventricular function in T-H injury through regulation of cellular energetic. The results suggest that the reduced SIRT1 levels in T-H injury may be related to declining mitochondrial function [94].

## 6. The Endothelial Protective Effect of Resveratrol in T-H Injury

Oxidative stress and superoxide radical generation are believed to contribute to the pathogenesis of endothelial dysfunction in low-flow states [95–97], resulting in inadequate tissue perfusion [96, 97].

Endothelial nicotinamide adenine dinucleotide phosphate-oxidase (NOX) is an important source of reactive oxygen species (ROS) of the vasculature, and, under various stressful conditions, a significant increase in NOX-generated ROS by the endothelium has been observed [95, 98]. Elevated ROS is a critical contributing factor to endothelial dysfunction, and antioxidants have been demonstrated to reduce ROS-induced injuries [95, 98]. Resveratrol has broad antioxidant and anti-inflammatory activities in a number of biological reactions [15, 99, 100], for instance, cardiovascular beneficial effects on atherosclerosis, ventricular arrhythmia, and myocardial ischemia-reperfusion I/R injury [101, 102]. Resveratrol's cardioprotective effects in I/R injury are achieved through its ROS-scavenging activity [77, 103]. However, the cardiovascular benefit of resveratrol may not simply be attributable to its antioxidant effect. Recent findings show that resveratrol reduces NOX activity in rat aortic endothelial cells and macrophages [20, 104, 105]. Resveratrol prevents T-H injury-induced oxidative stress and protects endothelium from subsequent oxidative functional damages [20]. The beneficial effects include inhibition of the NOX activity and direct scavenging of ROS. The protective effects of resveratrol are likely through suppression of the NOX enzyme complex activity in the cell membrane and the cytosol, including decreased membrane-bound proteins p22phox and gp91phox and cytosolic protein p47phox [20].

HO-1 appears to act as a protective agent in many organs against insults, such as trauma, ischemia, and oxidative stress [67, 106, 107]. Estrogen or flutamide enhances HO-1 expression, and resveratrol can modulate HO-1 induction via ER-related pathway [18, 20]. The upregulation in HO-1 is associated with the prevention of endothelial dysfunction and the salutary effects of resveratrol on endothelial function are mediated in part by upregulation of the HO-1-related pathway via ER [20].

## 7. Conclusions

Resveratrol has been shown to possess the beneficial effects in various studies and experimental conditions. There is increasing evidence that resveratrol maintains organ function after trauma or shock-like states. Resveratrol can attenuate organs injury in T-H injury through multiple pathways. However, the protective benefits of resveratrol may not simply be attributed to its anti-inflammatory or antioxidant effect. It is implicated that resveratrol is also mediated in part via a variety of intracellular signaling pathways, including the regulation of the HO-1/MAPK, PI3K/Akt, ER, and SIRT1. This complex network needs additional elucidation in future experimental studies and clinical trials.

## Figures and Tables

**Table 1:** The organ-protective effects and mechanisms of resveratrol in trauma-hemorrhagic injury.

Species	Target organ	Effective dose	Effects and mechanisms	Ref.
Sprague-Dawley rat	Liver	30 mg/kg/BW	Estrogen receptor-dependent HO-1 expression↑	[20]

Sprague-Dawley rat	Liver	30 mg/kg/BW	Reduction of T-H-induced proinflammatory parameters (CINC-1↓, CINC-3↓, ICAM-1, MPO↓, and IL-6↓); Akt-dependent HO-1 expression↑	[39]

Sprague-Dawley rat	Liver	30 mg/kg/BW	Reduction of T-H-induced proinflammatory parameters (CINC-1↓, CINC-3↓, ICAM-1, MPO↓, and IL-6↓); estrogen receptor-mediated pathway	[41]

Sprague-Dawley rat	Liver	30 mg/kg/BW	Reduction of T-H-induced mitochondria damage and hepatocyte injury; increase in SIRT1 expression; and decrease in p53 and NF-*κ*B activity; IL-6↓, MDA↓	[73]

Sprague-Dawley rat	Lung	30 mg/kg/BW	Reduction of T-H-induced proinflammatory parameters (CINC-1↓, CINC-3↓, ICAM-1, MPO↓, and IL-6↓)	[40]

Sprague-Dawley rat	Lung	30 mg/kg/BW	Estrogen receptor-dependent HO-1 expression↑	[20]

Sprague-Dawley rat	Intestine	30 mg/kg/BW	Reduction of T-H-induced proinflammatory parameters (CINC-1↓, CINC-3↓, ICAM-1, MPO↓, IL-6↓, and TNF-*α*↓); estrogen receptor-dependent P38/HO-1 expression↑	[18]

Sprague-Dawley rat	Heart	8 mg/kg/BW	Reduction of T-H-induced left ventricular contractility impairment through elevated SIRT1 expression; cardiac ATP↓, cytosolic cytochrome C↓, and plasma TNF-*α*↓	[86]

Sprague-Dawley rat	Heart	Not available	Reduction of T-H-induced mitochondria damage and improving left ventricular function through restored SIRT1 activity and PDK1 expression	[94]

Sprague-Dawley rat	Heart	30 mg/kg/BW	Reduction of T-H-induced proinflammatory parameters (ICAM-1↓, MPO↓, and IL-6↓); reduction of T-H-induced cardiac injury through elevated p-Akt activity	[38]

Sprague-Dawley rat	Endothelium	30 mg/kg/BW	Acetylcholine-induced endothelium-dependent relaxation↓ through estrogen receptor-dependent pathway; ROS radical/NADPH oxidase expression↓	[20]

Sprague-Dawley rat	Aorta	30 mg/kg/BW	NADPH-stimulated ROS↓; aortic p22phox, p47phox, gp91phox, NOX1, and NOX4 mRNA levels↓	[20]

Note: the species (Sprague-Dawley rat) are all the same in Table 1.

BW: body weight; ER: estrogen receptor; HO-1: hemeoxygenase-1; SIRT1: sirtuin 1; NF-*κ*B: nuclear factor-kappa B; MDA: malondialdehyde; TNF-*α*: tumor necrosis factor-alpha; CINC-1: cytokine-induced neutrophil chemoattractant 1; ICAM-1: intercellular adhesion molecule 1; MPO: myeloperoxidase; IL-6: interleukin 6; ROS: reactive oxygen species; NOX: NADPH oxidase; PDK1: pyruvate dehydrogenase kinase 1.

## Abbreviations

T-H: Trauma-hemorrhage
ER: Estrogen receptor
SIRT1: Sirtuin 1
HO-1: Hemeoxygenase-1
p38 MAPK: p38 mitogen-activated protein kinase
PI3K: Phosphatidylinositol 3-kinase
Akt: Protein kinase B
NF-*κ*B: Nuclear factor-kappa B
ROS: Reactive oxygen species
MDA: Malondialdehyde
NOX: NADPH oxidase
MPO: Myeloperoxidase
CINC-1: Cytokine-induced neutrophil chemoattractant 1
ICAM-1: Intercellular adhesion molecule 1
IL-6: Interleukin 6
TNF-*α*: Tumor necrosis factor-alpha
PDK1: Pyruvate dehydrogenase kinase 1.

## References

[1] Yu W., Fu Y. C., Wang W. (2012). Cellular and molecular effects of resveratrol in health and disease. *Journal of Cellular Biochemistry*.

[2] Chu L. M., Lassaletta A. D., Robich M. P., Sellke F. W. (2011). Resveratrol in the prevention and treatment of coronary artery disease. *Current Atherosclerosis Reports*.

[3] Hamza S. M., Dyck J. R. B. (2014). Systemic and renal oxidative stress in the pathogenesis of hypertension: modulation of long-term control of arterial blood pressure by resveratrol. *Frontiers in Physiology*.

[4] Song L., Chen L., Zhang X., Li J., Le W. (201). Resveratrol ameliorates motor neuron degeneration and improves survival in SOD1^G93A^ mouse model of amyotrophic lateral sclerosis. *BioMed Research International*.

[5] Yavuz S., Aydin N. E., Celik O., Yilmaz E., Ozerol E., Tanbek K. (2014). Resveratrol successfully treats experimental endometriosis through modulation of oxidative stress and lipid peroxidation. *Journal of Cancer Research and Therapeutics*.

[6] Andrade J. M. O., Paraíso A. F., de Oliveira M. V. M. (2014). Resveratrol attenuates hepatic steatosis in high-fat fed mice by decreasing lipogenesis and inflammation. *Nutrition*.

[7] Azachi M., Yatuv R., Katz A., Hagay Y., Danon A. (2014). A novel red grape cells complex: health effects and bioavailability of natural resveratrol. *International Journal of Food Sciences and Nutrition*.

[8] Heebøll S., Thomsen K. L., Pedersen S. B., Vilstrup H., George J., Grønbæk H. (2014). Effects of resveratrol in experimental and clinical non-alcoholic fatty liver disease. *World Journal of Hepatology*.

[9] Tian J., Chen J. W. (2012). The application of resveratrol in treating rheumatic disease: a review. *Zhongguo Zhong Xi Yi Jie He Za Zhi*.

[10] Legg K. (2012). Metabolic disease: identifying novel targets of resveratrol. *Nature Reviews Drug Discovery*.

[11] Magyar K., Halmosi R., Palfi A. (2012). Cardioprotection by resveratrol: a human clinical trial in patients with stable coronary artery disease. *Clinical Hemorheology and Microcirculation*.

[12] Li F., Gong Q., Dong H., Shi J. (2012). Resveratrol, a neuroprotective supplement for Alzheimer's disease. *Current Pharmaceutical Design*.

[13] Wood L. G., Wark P. A. B., Garg M. L. (2010). Antioxidant and anti-inflammatory effects of resveratrol in airway disease. *Antioxidants and Redox Signaling*.

[14] Csiszar A. (2011). Anti-inflammatory effects of resveratrol: possible role in prevention of age-related cardiovascular disease. *Annals of the New York Academy of Sciences*.

[15] Wang W., Sun L., Zhang P., Song J., Liu W. (2014). An anti-inflammatory cell-free collagen/resveratrol scaffold for repairing osteochondral defects in rabbits. *Acta Biomaterialia*.

[16] Nwachukwu J. C., Srinivasan S., Bruno N. E. (2014). Resveratrol modulates the inflammatory response via an estrogen receptor-signal integration network. *eLife*.

[17] Saleh M. C., Connell B. J., Saleh T. M. (2013). Resveratrol induced neuroprotection is mediated via both estrogen receptor subtypes, ER_*α*_ and ER_*β*_. *Neuroscience Letters*.

[18] Yu H.-P., Hwang T.-L., Hsieh P.-W., Lau Y.-T. (2011). Role of estrogen receptor-dependent upregulation of P38 MAPK/heme oxygenase 1 in resveratrol-mediated attenuation of intestinal injury after trauma-hemorrhage. *Shock*.

[19] Klinge C. M., Blankenship K. A., Risinger K. E. (2005). Resveratrol and estradiol rapidly activate MAPK signaling through estrogen receptors alpha and beta in endothelial cells. *The Journal of Biological Chemistry*.

[20] Yu H.-P., Hwang T.-L., Hwang T.-L., Yen C.-H., Lau Y.-T. (2010). Resveratrol prevents endothelial dysfunction and aortic superoxide production after trauma hemorrhage through estrogen receptor-dependent hemeoxygenase-1 pathway. *Critical Care Medicine*.

[21] Ren J., Fan C., Chen N., Huang J., Yang Q. (2011). Resveratrol pretreatment attenuates cerebral ischemic injury by upregulating expression of transcription factor Nrf2 and HO-1 in Rats. *Neurochemical Research*.

[22] Zheng Y., Liu Y., Ge J. (2010). Resveratrol protects human lens epithelial cells against H_2_O_2_- induced oxidative stress by increasing catalase, SOD-1, and HO-1 expression. *Molecular Vision*.

[23] Tamaki N., Orihuela-Campos R. C., Inagaki Y., Fukui M., Nagata T., Ito H.-O. (2014). Resveratrol improves oxidative stress and prevents the progression of periodontitis via the activation of the Sirt1/AMPK and the Nrf2/antioxidant defense pathways in a rat periodontitis model. *Free Radical Biology and Medicine*.

[24] Li J., Feng L., Xing Y. (2014). Radioprotective and antioxidant effect of resveratrol in hippocampus by activating Sirt1. *International Journal of Molecular Sciences*.

[25] Ren Z., Wang L., Cui J. (2013). Resveratrol inhibits NF-*κ*B signaling through suppression of p65 and IkappaB kinase activities. *Pharmazie*.

[26] Zhang J., Chen J., Yang J. (2013). Resveratrol attenuates oxidative stress induced by balloon injury in the rat carotid artery through actions on the ERK1/2 and NF-kappa B pathway. *Cellular Physiology and Biochemistry*.

[27] Mokni M., Hamlaoui S., Karkouch I. (2013). Resveratrol provides cardioprotection after ischemia/reperfusion injury via modulation of antioxidant enzyme activities. *Iranian Journal of Pharmaceutical Research*.

[28] Bagriyanik H. A., Ersoy N., Cetinkaya C. (2014). The effects of resveratrol on chronic constriction injury of sciatic nerve in rats. *Neuroscience Letters*.

[29] Zhang J., Tong N., Chen Y., Li P., Yang S., Zhao X. (2013). Resveratrol protects against vinorelbine-induced vascular endothelial cell injury. *Toxicology Mechanisms and Methods*.

[30] Zhang H. X., Duan G. L., Wang C. N., Zhang Y. Q., Zhu X. Y., Liu Y. J. (2014). Protective effect of resveratrol against endotoxemia-induced lung injury involves the reduction of oxidative/nitrative stress. *Pulmonary Pharmacology & Therapeutics*.

[31] Vin A. P., Hu H., Zhai Y. (2013). Neuroprotective effect of resveratrol prophylaxis on experimental retinal ischemic injury. *Experimental Eye Research*.

[32] Tunali-Akbay T., Sehirli O., Ercan F., Sener G. (2010). Resveratrol protects against methotrexate-induced hepatic injury in rats. *Journal of Pharmacy and Pharmaceutical Sciences*.

[33] de Jesus Soares T., Volpini R. A., Francescato H. D. C., Costa R. S., da Silva C. G. A., Coimbra T. M. (2007). Effects of resveratrol on glycerol-induced renal injury. *Life Sciences*.

[34] Gatson J. W., Liu M. M., Abdelfattah K. (2013). Resveratrol decreases inflammation in the brain of mice with mild traumatic brain injury. *Journal of Trauma and Acute Care Surgery*.

[35] McGhan L. J., Jaroszewski D. E. (2012). The role of toll-like receptor-4 in the development of multi-organ failure following traumatic haemorrhagic shock and resuscitation. *Injury*.

[36] Stein D. M., Menaker J., McQuillan K., Handley C., Aarabi B., Scalea T. M. (2010). Risk factors for organ dysfunction and failure in patients with acute traumatic cervical spinal cord injury. *Neurocritical Care*.

[37] Liu F.-C., Tsai Y.-F., Yu H.-P. (2013). Sirtinol attenuates trauma hemorrhage-induced hepatic injury through Akt-dependent pathway in rats. *Journal of Trauma and Acute Care Surgery*.

[38] Tsai Y.-F., Liu F.-C., Lau Y.-T., Yu H.-P. (2012). Role of Akt-dependent pathway in resveratrol-mediated cardioprotection after trauma-hemorrhage. *Journal of Surgical Research*.

[39] Yu H.-P., Yang S.-C., Lau Y.-T., Hwang T.-L. (2010). Role of Akt-dependent up-regulation of hemeoxygenase-1 in resveratrol-mediated attenuation of hepatic injury after trauma hemorrhage. *Surgery*.

[40] Wu C.-T., Yu H.-P., Chung C.-Y., Lau Y.-T., Liao S.-K. (2008). Attenuation of lung inflammation and pro-inflammatory cytokine production by resveratrol following trauma-hemorrhage. *The Chinese Journal of Physiology*.

[41] Yu H.-P., Hsu J.-C., Hwang T.-L., Yen C.-H., Lau Y.-T. (2008). Resveratrol attenuates hepatic injury after trauma-hemorrhage via estrogen receptor-related pathway. *Shock*.

[42] Liu F.-C., Day Y.-J., Liao C.-H., Liou J.-T., Mao C.-C., Yu H.-P. (2009). Hemeoxygenase-1 upregulation is critical for sirtinol-mediated attenuation of lung injury after trauma-hemorrhage in a rodent model. *Anesthesia & Analgesia*.

[43] Yu H.-P., Choudhry M. A., Shimizu T. (2006). Mechanism of the salutary effects of flutamide on intestinal myeloperoxidase activity following trauma-hemorrhage: up-regulation of estrogen receptor-*β*-dependent HO-1. *Journal of Leukocyte Biology*.

[44] Sonnier D. I., Makley A. T., Friend L. A. W., Bailey S. R., Lentsch A. B., Pritts T. A. (2011). Hemorrhagic shock induces a proinflammatory milieu in the gut lumen. *Journal of Surgical Research*.

[45] Shimizu T., Yu H.-P., Hsieh Y.-C. (2007). Flutamide attenuates pro-inflammatory cytokine production and hepatic injury following trauma-hemorrhage via estrogen receptor-related pathway. *Annals of Surgery*.

[46] Suzuki T., Shimizu T., Yu H.-P. (2007). Tissue compartment-specific role of estrogen receptor subtypes in immune cell cytokine production following trauma-hemorrhage. *Journal of Applied Physiology*.

[47] Yu H.-P., Shimizu T., Hsieh Y.-C. (2006). Tissue-specific expression of estrogen receptors and their role in the regulation of neutrophil infiltration in various organs following trauma-hemorrhage. *Journal of Leukocyte Biology*.

[48] Liu F.-C., Yu H.-P., Hwang T.-L., Tsai Y.-F. (2012). Protective effect of tropisetron on rodent hepatic injury after trauma-hemorrhagic shock through P38 MAPK-dependent hemeoxygenase-1 expression. *PLoS ONE*.

[49] Liu F.-C., Day Y.-J., Liou J.-T., Lau Y.-T., Yu H.-P. (2008). Sirtinol attenuates hepatic injury and pro-inflammatory cytokine production following trauma-hemorrhage in male Sprague-Dawley rats. *Acta Anaesthesiologica Scandinavica*.

[50] Yu H.-P., Hsieh P.-W., Chang Y.-J., Chung P.-J., Kuo L.-M., Hwang T.-L. (2009). DSM-RX78, a new phosphodiesterase inhibitor, suppresses superoxide anion production in activated human neutrophils and attenuates hemorrhagic shock-induced lung injury in rats. *Biochemical Pharmacology*.

[51] Yu H.-P., Yang S., Hsieh Y.-C., Choudhry M. A., Bland K. I., Chaudry I. H. (2006). Maintenance of lung myeloperoxidase activity in proestrus females after trauma-hemorrhage: upregulation of heme oxygenase-1. *The American Journal of Physiology—Lung Cellular and Molecular Physiology*.

[52] Dayal S. D., Haskó G., Lu Q. (2002). Trauma/hemorrhagic shock mesenteric lymph upregulates adhesion molecule expression and IL-6 production in human umbilical vein endothelial cells. *Shock*.

[53] Yang R., Han X., Uchiyama T. (2003). IL-6 is essential for development of gut barrier dysfunction after hemorrhagic shock and resuscitation in mice. *The American Journal of Physiology: Gastrointestinal and Liver Physiology*.

[54] Meng Z. H., Dyer K., Billiar T. R., Tweardy D. J. (2001). Essential role for IL-6 in postresuscitation inflammation in hemorrhagic shock. *American Journal of Physiology—Cell Physiology*.

[55] Suzuki T., Shimizu T., Yu H.-P. (2007). Estrogen receptor-alpha predominantly mediates the salutary effects of 17beta-estradiol on splenic macrophages following trauma-hemorrhage. *The American Journal of Physiology: Cell Physiology*.

[56] Yu H.-P., Pang S.-T., Chaudry I. H. (2013). Hepatic gene expression patterns following trauma-hemorrhage: effect of posttreatment with estrogen. *Shock*.

[57] Sebai H., Sani M., Yacoubi M. T., Aouani E., Ghanem-Boughanmi N., Ben-Attia M. (2010). Resveratrol, a red wine polyphenol, attenuates lipopolysaccharide-induced oxidative stress in rat liver. *Ecotoxicology and Environmental Safety*.

[58] Choudhry M. A., Bland K. I., Chaudry I. H. (2007). Trauma and immune response—effect of gender differences. *Injury*.

[59] Choudhry M. A., Schwacha M. G., Hubbard W. J. (2005). Gender differences in acute response to trauma-hemorrhage. *Shock*.

[60] Shimizu T., Yu H.-P., Suzuki T. (2007). The role of estrogen receptor subtypes in ameliorating hepatic injury following trauma-hemorrhage. *Journal of Hepatology*.

[61] Suzuki T., Shimizu T., Yu H.-P., Hsieh Y.-C., Choudhry M. A., Chaudry I. H. (2007). Salutary effects of 17beta-estradiol on T-cell signaling and cytokine production after trauma-hemorrhage are mediated primarily via estrogen receptor-alpha. *The American Journal of Physiology: Cell Physiology*.

[62] Yu H.-P., Chaudry I. H. (2009). The role of estrogen and receptor agonists in maintaining organ function after trauma-hemorrhage. *Shock*.

[63] Yu H. P., Hsieh Y. C., Suzuki T. (2006). Salutary effects of estrogen receptor-*β* agonist on lung injury after trauma-hemorrhage. *The American Journal of Physiology—Lung Cellular and Molecular Physiology*.

[64] Yu H. P., Hsieh Y. C., Suzuki T. (2007). The PI3K/Akt pathway mediates the nongenomic cardioprotective effects of estrogen following trauma-hemorrhage. *Annals of Surgery*.

[65] Elmarakby A. A., Faulkner J., Baban B., Sullivan J. C. (2012). Induction of hemeoxygenase-1 reduces renal oxidative stress and inflammation in diabetic spontaneously hypertensive rats. *International Journal of Hypertension*.

[66] Elmarakby A. A., Faulkner J., Posey S. P., Sullivan J. C. (2010). Induction of hemeoxygenase-1 attenuates the hypertension and renal inflammation in spontaneously hypertensive rats. *Pharmacological Research*.

[67] Hsu J.-T., Kan W.-H., Hsieh C.-H., Choudhry M. A., Bland K. I., Chaudry I. H. (2009). Mechanism of salutary effects of estrogen on cardiac function following trauma-hemorrhage: akt-dependent HO-1 up-regulation. *Critical Care Medicine*.

[68] Hsu J.-T., Kan W. H., Hsieh C.-H. (2007). Mechanism of estrogen-mediated attenuation of hepatic injury following trauma-hemorrhage: Akt-dependent HO-1 up-regulation. *Journal of Leukocyte Biology*.

[69] Hsu J.-T., Yeh H.-C., Chen T.-H. (2013). Role of Akt/HO-1 pathway in estrogen-mediated attenuation of trauma-hemorrhage-induced lung injury. *Journal of Surgical Research*.

[70] Liu F.-C., Hwang T.-L., Lau Y.-T., Yu H.-P. (2011). Mechanism of salutary effects of astringinin on rodent hepatic injury following trauma-hemorrhage: Akt-dependent hemeoxygenase-1 signaling pathways. *PLoS ONE*.

[71] Chen B., Xue J., Meng X., Slutzky J. L., Calvert A. E., Chicoine L. G. (2014). Resveratrol prevents hypoxia-induced arginase II expression and proliferation of human pulmonary artery smooth muscle cells via Akt-dependent signaling. *The American Journal of Physiology—Lung Cellular and Molecular Physiology*.

[72] Simão F., Matté A., Pagnussat A. S., Netto C. A., Salbego C. G. (2012). Resveratrol prevents CA1 neurons against ischemic injury by parallel modulation of both GSK-3beta and CREB through PI3-K/Akt pathways. *European Journal of Neuroscience*.

[73] Powell R. D., Swet J. H., Kennedy K. L., Huynh T. T., McKillop I. H., Evans S. L. (2014). Resveratrol attenuates hypoxic injury in a primary hepatocyte model of hemorrhagic shock and resuscitation. *Journal of Trauma and Acute Care Surgery*.

[74] Liu F.-C., Liu F.-W., Yu H.-P. (2011). Ondansetron attenuates hepatic injury via p38 MAPK-dependent pathway in a rat haemorrhagic shock model. *Resuscitation*.

[75] Hsu J.-T., Kan W.-H., Hsieh C.-H. (2008). Mechanism of estrogen-mediated intestinal protection following trauma-hemorrhage: p38 MAPK-dependent upregulation of HO-1. *The American Journal of Physiology—Regulatory Integrative and Comparative Physiology*.

[76] Liu X. W., Ji E. F., He P., Xing R. X., Tian B. X., Li X. D. (2014). Protective effects of the p38 MAPK inhibitor SB203580 on NMDA-induced injury in primary cerebral cortical neurons. *Molecular Medicine Reports*.

[77] Wang S., Huang Q., Guo J. (2014). Local thermal injury induces general endothelial cell contraction through p38 MAP kinase activation. *Acta Pathologica, Microbiologica et Immunologica Scandinavica*.

[78] Ma J., Fang Y.-Q., Gu A.-M., Wang F.-F., Zhang S., Li K.-C. (2013). P38 activation is more important than ERK activation in lung injury induced by prolonged hyperbaric oxygen. *Undersea and Hyperbaric Medicine*.

[79] Shifflett D. E., Jones S. L., Moeser A. J., Blikslager A. T. (2004). Mitogen-activated protein kinases regulate COX-2 and mucosal recovery in ischemic-injured porcine ileum. *The American Journal of Physiology: Gastrointestinal and Liver Physiology*.

[80] Aoudjit L., Stanciu M., Li H., Lemay S., Takano T. (2003). p38 mitogen-activated protein kinase protects glomerular epithelial cells from complement-mediated cell injury. *The American Journal of Physiology—Renal Physiology*.

[81] Sánchez-Fidalgo S., Cárdeno A., Villegas I., Talero E., de la Lastra C. A. (2010). Dietary supplementation of resveratrol attenuates chronic colonic inflammation in mice. *European Journal of Pharmacology*.

[82] Lv X. C., Zhou H. Y. (2012). Resveratrol protects H9c2 embryonic rat heart derived cells from oxidative stress by inducing autophagy: role of p38 mitogen-activated protein kinase. *Canadian Journal of Physiology and Pharmacology*.

[83] Babu D., Soenen S. J., Raemdonck K. (2012). TNF-*α*/cycloheximide-induced oxidative stress and apoptosis in murine intestinal epithelial MODE-K cells. *Current Pharmaceutical Design*.

[84] Mokni M., Limam F., Elkahoui S., Amri M., Aouani E. (2007). Strong cardioprotective effect of resveratrol, a red wine polyphenol, on isolated rat hearts after ischemia/reperfusion injury. *Archives of Biochemistry and Biophysics*.

[85] Hung L.-M., Su M.-J., Chen J.-K. (2004). Resveratrol protects myocardial ischemia-reperfusion injury through both NO-dependent and NO-independent mechanisms. *Free Radical Biology and Medicine*.

[86] Jian B., Yang S., Chaudry I. H., Raju R. (2012). Resveratrol improves cardiac contractility following trauma-hemorrhage by modulating Sirt1. *Molecular Medicine*.

[87] Jiang X., Ai C., Shi E., Nakajima Y., Ma H. (2009). Neuroprotection against spinal cord ischemia-reperfusion injury induced by different ischemic postconditioning methods: roles of phosphatidylinositol 3-kinase-akt and extracellular signal-regulated kinase. *Anesthesiology*.

[88] Lv L., Meng Q., Xu J., Gong J., Cheng Y., Jiang S. (2012). Ligustrazine attenuates myocardial ischemia reperfusion injury in rats by activating the phosphatidylinositol 3-kinase/Akt pathway. *Annals of Clinical and Laboratory Science*.

[89] Schabbauer G., Tencati M., Pedersen B., Pawlinski R., Mackman N. (2004). PI3K-Akt pathway suppresses coagulation and inflammation in endotoxemic mice. *Arteriosclerosis, Thrombosis, and Vascular Biology*.

[90] Fung-Leung W.-P. (2011). Phosphoinositide 3-kinase delta (PI3K*δ*) in leukocyte signaling and function. *Cellular Signalling*.

[91] He H., Yu F. X., Sun C., Luo Y. (2011). CBP/p300 and SIRT1 are involved in transcriptional regulation of S-phase specific histone genes. *PLoS ONE*.

[92] Pruitt K., Zinn R. L., Ohm J. E. (2006). Inhibition of SIRT1 reactivates silenced cancer genes without loss of promoter DNA hypermethylation. *PLoS Genetics*.

[93] Li Y.-G., Zhu W., Tao J.-P. (2013). Resveratrol protects cardiomyocytes from oxidative stress through SIRT1 and mitochondrial biogenesis signaling pathways. *Biochemical and Biophysical Research Communications*.

[94] Jian B., Yang S., Chaudry I. H., Raju R. (2014). Resveratrol restores sirtuin 1 (SIRT1) activity and pyruvate dehydrogenase kinase 1 (PDK1) expression after hemorrhagic injury in a rat model. *Molecular Medicine*.

[95] Yu H.-P., Lui P.-W., Hwang T.-L., Yen C.-H., Lau Y.-T. (2006). Propofol improves endothelial dysfunction and attenuates vascular superoxide production in septic rats. *Critical Care Medicine*.

[96] Ba Z. F., Kuebler J. F., Rue L. W., Bland K. I., Wang P., Chaudry I. H. (2003). Gender dimorphic tissue perfusion response after acute hemorrhage and resuscitation: role of vascular endothelial cell function. *American Journal of Physiology: Heart and Circulatory Physiology*.

[97] Wang P., Ba Z. F., Chaudry I. H. (1993). Endothelial cell dysfunction occurs very early following trauma- hemorrhage and persists despite fluid resuscitation. *The American Journal of Physiology: Heart and Circulatory Physiology*.

[98] Montezano A. C., Touyz R. M. (2012). Reactive oxygen species and endothelial function—role of nitric oxide synthase uncoupling and nox family nicotinamide adenine dinucleotide phosphate oxidases. *Basic & Clinical Pharmacology and Toxicology*.

[99] Orsu P., Murthy B. V. S. N., Akula A. (2013). Cerebroprotective potential of resveratrol through anti-oxidant and anti-inflammatory mechanisms in rats. *Journal of Neural Transmission*.

[100] Bo S., Ciccone G., Castiglione A. (2013). Anti-inflammatory and antioxidant effects of resveratrol in healthy smokers a randomized, double-blind, placebo-controlled, cross-over trial. *Current Medicinal Chemistry*.

[101] Petrovski G., Gurusamy N., Das D. K. (2011). Resveratrol in cardiovascular health and disease. *Annals of the New York Academy of Sciences*.

[102] Toklu H. Z., Şehirli Ö., Erşahin M. (2010). Resveratrol improves cardiovascular function and reduces oxidative organ damage in the renal, cardiovascular and cerebral tissues of two-kidney, one-clip hypertensive rats. *Journal of Pharmacy and Pharmacology*.

[103] Cong X., Li Y., Lu N. (2014). Resveratrol attenuates the inflammatory reaction induced by ischemia/reperfusion in the rat heart. *Molecular Medicine Reports*.

[104] Tang Y., Xu J., Qu W. (2012). Resveratrol reduces vascular cell senescence through attenuation of oxidative stress by SIRT1/NADPH oxidase-dependent mechanisms. *The Journal of Nutritional Biochemistry*.

[105] Chow S.-E., Hshu Y.-C., Wang J.-S., Chen J.-K. (2007). Resveratrol attenuates oxLDL-stimulated NADPH oxidase activity and protects endothelial cells from oxidative functional damages. *Journal of Applied Physiology*.

[106] Wang J., Hu X., Jiang H. (2014). Nrf-2-HO-1-HMGB1 axis: an important therapeutic approach for protection against myocardial ischemia and reperfusion injury. *International Journal of Cardiology*.

[107] Tsuchihashi S., Zhai Y., Fondevila C., Busuttil R. W., Kupiec-Weglinski J. W. (2005). HO-1 upregulation suppresses type 1 IFN pathway in hepatic ischemia/reperfusion injury. *Transplantation Proceedings*.

